# Sweet Taste Receptors Mediated ROS-NLRP3 Inflammasome Signaling Activation: Implications for Diabetic Nephropathy

**DOI:** 10.1155/2018/7078214

**Published:** 2018-02-20

**Authors:** Luping Zhou, Wei Huang, Youhua Xu, Chenlin Gao, Ting Zhang, Man Guo, Yan Liu, Jingya Ding, Ludan Qin, Zihao Xu, Yang Long, Yong Xu

**Affiliations:** ^1^Department of Endocrinology, Affiliated Hospital of Southwest Medical University, Luzhou, Sichuan 646000, China; ^2^The Graduate School of Southwest Medical University, Luzhou, Sichuan 646000, China; ^3^Faculty of Chinese Medicine, Macau University of Science and Technology, Avenida Wai Long, Taipa, Macau, China; ^4^State Key Laboratory of Quality Research in Chinese Medicine, Macau University of Science and Technology, Avenida Wai Long, Taipa, Macau, China; ^5^Key Laboratory of Medical Electrophysiology of Ministry of Education, Collaborative Innovation Center for Prevention and Treatment of Cardiovascular Disease of Sichuan Province, Southwest Medical University, Luzhou, Sichuan 646000, China

## Abstract

Previous studies demonstrated that ROS-NLRP3 inflammasome signaling activation was involved in the pathogenesis of diabetic nephropathy (DN). Recent research has shown that sweet taste receptors (STRs) are important sentinels of innate immunity. Whether high glucose primes ROS-NLRP3 inflammasome signaling via STRs is unclear. In this study, diabetic mouse model was induced by streptozotocin (STZ) in vivo; mouse glomerular mesangial cells (GMCs) and human proximal tubular cells were stimulated by high glucose (10, 20, and 30 mmol/L) in vitro; STR inhibitor lactisole was used as an intervention reagent to evaluate the role and mechanism of the STRs in the pathogenesis of DN. Our results showed that the expression of STRs and associated signaling components (G*α*-gustducin, PLC*β*2, and TRPM5) was obviously downregulated under the condition of diabetes in vivo and in vitro. Furthermore, lactisole significantly mitigated the production of intracellular ROS and reversed the high glucose-induced decrease of Ca^2+^ and the activation of NLRP3 inflammasome signaling in vitro (*p* < 0.05). These combined results support the hypothesis that STRs could be involved in the activation of ROS-NLRP3 inflammasome signaling in the pathogenesis of DN, suggesting that STRs may act as new therapeutic targets of DN.

## 1. Introduction

Oxidative stress and persistent microinflammatory state in circulatory and renal tissues play a key role in the development and progression of diabetic nephropathy (DN) [1, 2]. The NOD-like receptor pyrin 3 (NLRP3) inflammasome is an intracellular platform that recruits the adaptor molecule-apoptosis-associated speck-like protein (ASC) by pyrin domain, and then ASC hydrolyzes procaspase-1, and finally, active caspase-1 cleaves pro-IL-1*β* into its mature form in response to “danger” signals [3]. Therefore, the pharmacological targeting of the oxygen species- (ROS-) NLRP3-mediated inflammatory response may help with the design of a new approach to develop therapeutic strategies for preventing the deterioration of kidney injury in the pathogenesis of DN. However, the mechanism mediating NLRP3 inflammasome activation in DN has not been completely clarified yet [4].

Among five basic taste qualities, sweet taste permits the identification of energy-rich nutrients, which are very attractive for animals and influence food intake [5, 6]. Studies have revealed two classes of GPCRs that have been identified, including taste 1 receptor family (T1R) and the taste 2 receptor family (T2R), and two subtypes of the T1R family, including T1R member 2 (T1R2) and T1R member 3 (T1R3), form heterodimers to act as sweet taste receptors (STRs), which are expressed in taste buds and in the gastrointestinal tract and respond to various chemically distinct compounds, such as natural sugars, noncaloric artificial and natural sweeteners, some D-amino acids, and sweet-tasting proteins [7]. As a natural ligand for STRs, glucose binding to STRs leads to activation of the heteromeric G*α*-gustducin. Phospholipase C *β*2 (PLC*β*2) is subsequently stimulated, leading to release of intracellular Ca^2+^ and activation of the transient receptor potential channel M5 (TRPM5). Finally, taste cells are depolarized, generate action potentials, and release transmitters (ATP) via pannexin-1 hemichannels [8].

Accumulating evidence shows that STRs are ubiquitous throughout the body, including the stomach, pancreas, gut, liver, and brain. These STRs are heavily involved in nutrient sensing, monitoring changes in energy stores, and triggering metabolic and behavioral responses to maintain energy balance and immune response [9–11]. Recent study indicated that sweet taste disorder in patients with abnormal glucose tolerance was associated with increased sugar intake and vascular complications, including DN and diabetic retinopathy [12]. However, whether STRs are also expressed in renal tissue and take part in the activation of ROS-NLRP3 inflammasome signaling under high-glucose stress is unclear. In this study, we detected the expression of T1R2, T1R3, G*α*-gustducin, PLC*β*2, TRPM5, NLRP3, procaspase-1, and IL-1*β* in renal tissue of DN model mouse and renal innate cells (mouse GMCs and human proximal tubular cells (HK-2)) under high-glucose stress and then observed the changes of these players using lactisole-blocked STRs, aiming to elucidate the role of STRs in the activation of ROS-NLRP3 inflammasome signaling in the pathogenesis of DN.

## 2. Materials and Methods

### 2.1. Establishing the Animal Model and Sample Collection

Male C57BL/6 mouse weighing 20 g was purchased from the Biotechnology Corporation of Dashuo (Chengdu, China). The mouse was randomly allocated into two groups: a control group (NC group, *n* = 10) and a diabetes mellitus group (DM group, *n* = 10). Mouse in the DM group was rendered diabetic by intraperitoneal injection of Streptozocin (STZ, Sigma, USA), which was dissolved in 0.1 M citrate buffer at pH 4.5, at a dose of 40 mg/kg for 4 days. Meanwhile, a mouse in the NC group received an intraperitoneal injection of the same volume of citrate buffer. After 4 days following the STZ injection, fasting blood glucose (FBG) measurements were performed in blood samples from tail veins, and FBG levels of ≥16.7 mmol/L lasting 3 days were confirmed as being “diabetic.”

All mice were weighed and 24-hour urinary microalbumin (mAlb) was collected in metabolic cages (Hattera, USA) every two weeks. Urinary protein and urinary creatinine concentrations were measured according to the manufacturers' procedures described in the kits (AfinionTM ACR, Axis-Shield PoC AS, Norway), and urine albumin-creatinine ratios (ACR) were calculated. After 12 weeks, all mice were sacrificed and heart blood was collected to measure blood urea nitrogen (BUN) levels by automatic biochemistry analyzer. Both kidneys were cut along the coronal plane; the right kidneys were used for pathology, and the left renal tissues were preserved in RNA later solution (Thermo Fisher Scientific, USA) until required for qRT-PCR.

### 2.2. Cell Culture, Stimulation, and Viability Assay

Mouse GMCs (SV40 MES 13) and human proximal tubular cells (HK-2) were purchased from the China Center for Type Culture Collection (CCTCC) and were cultured in Dulbecco's modified Eagle's medium (DMEM, Glbco) containing 5.6 mmol/L glucose and 10% fetal bovine serum (FBS, Glbco) at 37°C and 5% CO_2._

Initially, to determine proper concentrations of high glucose and lactisole, cells were randomly divided into the following groups: (1) normal control group (NC group, 5.6 mmol/L glucose); (2) high-glucose treatment group (HG group; cells were treated with culture medium containing 10 mmol/L, 20 mmol/L, and 30 mmol/L glucose); (3) osmotic pressure group (OP group, with medium that contained 5.6 mmol/L glucose + 24.4 mmol/L mannitol); (4) lactisole (Sigma-Aldrich, MO, USA) at 3, 5, and 10 mmol/L as an inhibitor of STR intervention group.

The cells in each group were induced for 6, 12, or 24 h before the culture supernatant was collected, and the protein and mRNA were extracted for further study. Viability of GMCs was determined by 3-(4,5-dimethylthiazol-2-yl)-2,5-diphenyltetrazolium bromide (MTT). For MTT analysis, absorbance at 570 nm was measured for the experimental groups using a microplate reader according to the manufacturer's instruction. Each experiment was performed in duplicate and repeated thrice.

### 2.3. Immunohistochemistry and Immunofluorescence Staining

Sections were incubated with the following primary antibodies: anti-T1R2 (goat polyclonal antibody; 1 : 100 dilution; Santa Cruz, USA), anti-T1R3 (goat polyclonal antibody; 1 : 100 dilution; Santa Cruz, USA), anti-NLRP3 (rabbit monoclonal antibody; 1 : 100 dilution; CST, USA), and anti-IL-1*β* (rabbit monoclonal antibody; 1 : 100 dilution; CST, USA) overnight at 4°C. After sections were washed with PBS, they were incubated with horseradish peroxidase (HRP) or fluorescein isothiocyanate fluorescent dye-conjugated secondary antibodies (1 : 200 dilution, Biosynthesis Biotech, China) for 2 h at room temperature. For visualizing the signals, sections were treated with peroxidase substrate DAB (3,3-diaminobenzidine) and counterstained with hematoxylin.

GMCs were grown on coverslips in 6-well plates. After overnight adherence, cells were incubated with different compounds for 24 h as described above and then were fixed in 4% paraformaldehyde and then blocked with 5% rabbit serum. The cells were incubated overnight with the anti-NLRP3, caspase-1, and IL-1*β* antibody (1 : 100 dilution). Images were taken with a fluorescence microscope (Leica, Germany), and the values of semiquantitative analysis for average intensity were assessed by Image-Pro Plus 6.0 software.

### 2.4. Protein Extraction and Western Blotting

Total protein was extracted from kidney, mouse GMCs, and human proximal tubular cells using a protein extraction kit (Kaiji, Shanghai, China). Proteins were separated by sodium dodecyl sulfate-polyacrylamide gel electrophoresis and transferred to a polyvinylidene difluoride (PVDF) membrane (Millipore). Immunoblotting was performed using anti-T1R2 antibody (1 : 800 dilution), anti-T1R3 antibody (1 : 1000 dilution), anti-NLRP3 antibody (1 : 800 dilution), anti-procaspase-1 antibody (rabbit monoclonal antibody; 1 : 800 dilution; CST, USA), anti-IL-1*β* antibody (1 : 800 dilution), and anti-GAPDH antibody (mouse; 1 : 800 dilution; Beyotime, China).

### 2.5. RNA Extraction and Quantitative Real-Time PCR (qRT-PCR) Analysis

Total RNA was isolated from kidney and GMCs using an RNA extraction kit (ComWin Biotech, Beijing, China). The isolated RNA was subjected to reverse transcription using the PrimeScript RT Reagent Kit (TaKaRa). The synthesized cDNA was used as a template for the quantitative PCR analysis. The house-keeping gene *β*-actin was quantified as an internal control. The primer sequences for all studied genes are listed in Table 1. qRT-PCR was performed on 7900HT Fast Real-Time PCR system (Thermo Fisher Scientific, USA). The thermal cycling program used was as follows: an initial step at 95°C for 10 minutes, followed by 40 cycles of denaturation at 95°C for 15 seconds, annealing at 60°C for 34 seconds, and extension at 72°C for 15 seconds. The melting curve of each PCR product was obtained by continuous fluorescence monitoring at a temperature gradient ramp from 60 to 95°C. The quantitative PCR reactions were performed in triplicate to remove any outliers. The relative changes of target genes were analyzed by the 2^-ΔΔCT^ method.

### 2.6. Detection of Intracellular ROS Level

Intracellular production of ROS was measured using the ROS assay kit (Beyotime Institute of Biotechnology, China) with oxidation of 2′,7′-dichlorofluorescin diacetate (DCFH-DA) to fluorescent 2′,7′-dichlorofluorescin (DCF). The fluorescence images were taken with a fluorescence microscope (Leica, Germany) and measured in a plate reader with excitation at 488 nm and emission at 525 nm according to the manufacturer's instructions. The values were expressed as the mean absorbance normalized to the percentage of the normal control.

### 2.7. Enzyme-Linked Immunosorbent Assay (ELISA)

IL-1*β* protein level in the cells culture supernatant was determined using commercially available ELISA IL-1*β* kits (NeoBioscience, China) according to the manufacturer's protocols. IL-1*β* protein levels were determined by comparing the samples to the standard curve generated by the kit.

### 2.8. Ca^2+^ Measurement

GMCs were treated with 30 mmol/L glucose alone or were intervened by 5 mmol/L lactisole following 30 mmol/L glucose for 24 h, respectively. For Ca^2+^ imaging experiments, GMCs on coverslips were incubated with Fura-PE3 AM (1 *μ*M) for 30 min at 37°C in Ca^2+^-free standard bath solution. The ratio (F_340_/F_380_) of Ca^2+^ dye fluorescence was measured by a Nikon Ti-E system with NIS-Elements software. All the experiments were performed at room temperature (23–26°C).

### 2.9. Data Analysis

All data obtained from at least three independent experiments were expressed as the means ± standard deviation (SD), and between-group comparisons were analyzed using one-way analysis of variance (ANOVA), followed by the LSD post hoc test for multiple comparisons (SPSS 17 software). *p* < 0.05 was considered significant.

## 3. Results

### 3.1. STZ-Induced Changes in ACR and Renal Function of Diabetic Mouse

**Figure 1:**
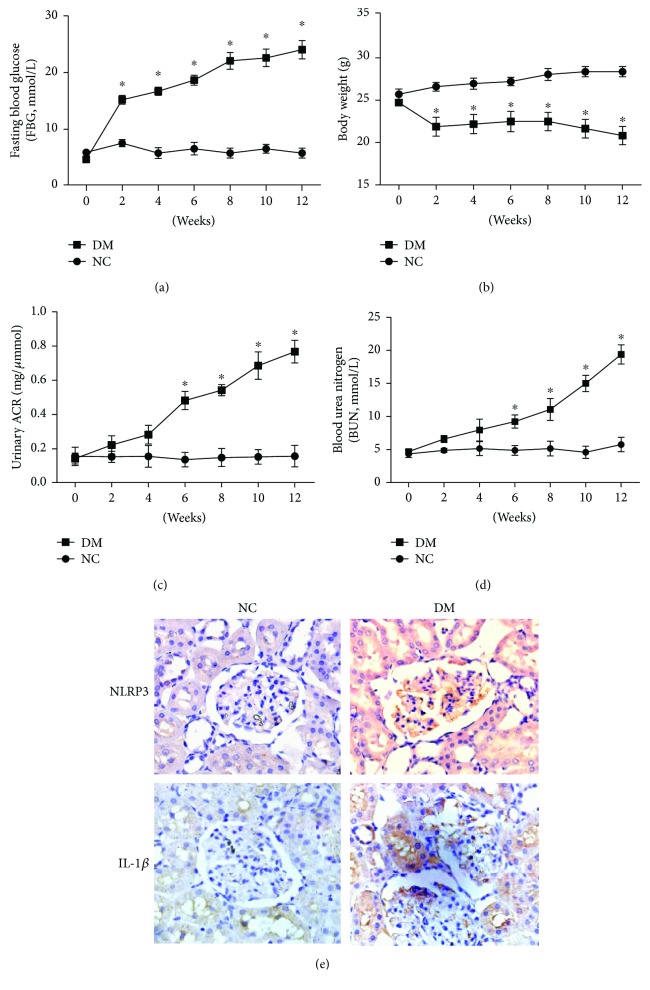
STZ-induced changes in FBG, body weight, ACR, and renal function of diabetic mouse. Male C57BL/6 mice were divided into diabetes mellitus group (DM group, *n* = 10) and normal control group (NC group, *n* = 10). Every two weeks, fasting blood glucose (a), body weight (b), ACR (c), and BUN (d) levels were examined. (e) STZ-induced expression of NLRP3 and IL-1*β* in renal tissue was observed by immunohistochemistry and light microscopy (200×). Data are presented as means ± SD. ^∗^*p* < 0.05 versus NC group at the same treatment time.

As shown in Figure 1, compared with the NC group, FBG (Figure 1(a)) levels were increased in the DM group (*p* < 0.05), but the body weight (Figure 1(b)) was significantly decreased (*p* < 0.05). More importantly, urinary ACR (Figure 1(c)) and serum BUN (Figure 1(d)) levels, as important features of DN, were increased in the DM groups (*p* < 0.05). The immunohistochemical stainings showed that there were increased NLRP3 and IL-1*β* expressions in renal tissue of DM group (Figure 1(e)). These data suggest that DN mouse models were successfully achieved by using STZ.

### 3.2. The Expression of STRs and Associated Signaling Components Was Downregulated in Renal Tissue of DM Mouse Models

**Figure 2:**
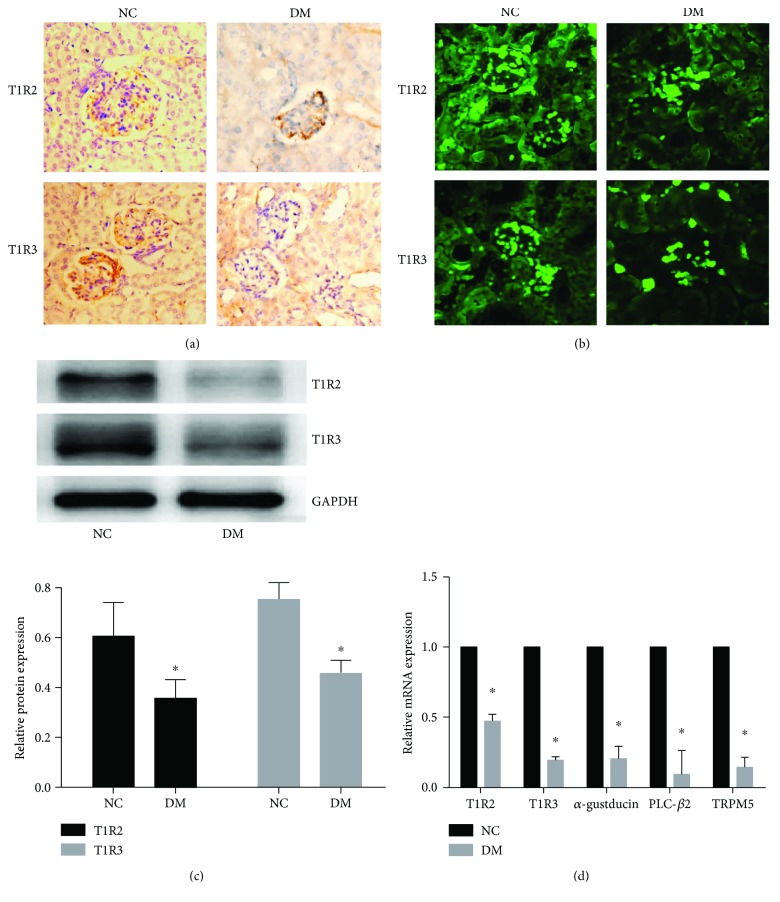
The expression of STRs and associated signaling components was downregulated in renal tissue of DM mouse models. Representative images of immunohistochemistry (a) and immunofluorescence (b) staining showed that T1R2 and T1R3 expression was downregulated in renal tissue of DM mouse models. T1R2 and T1R3 expression was represented as the positive yellow-brown stained area (immunohistochemistry, 200×) or green fluorescence area (immunofluorescence, 200×). (c) STR-associated signaling component (*α*-gustducin, PLC*β*2, and TRPM5) mRNA expression in renal tissues was detected by qRT-PCR. Data are presented as means ± SD; the gray graph confirmed these trends. ^∗^*p* < 0.05 versus NC group.

The expression of T1R2 and T1R3 in renal tissue was detected by immunohistochemistry (Figure 2(a)), immunofluorescence (Figure 2(b)), and Western blotting (Figure 2(c)). Compared with the NC group, these receptor molecular expression was downregulated in DM group (*p* < 0.05). Consistent with the expression of T1R2 and T1R3, the mRNA expression of STR signaling molecules, G*α*-gustducin, PLC*β*2, and TRPM5 was also inhibited in DM group (*p* < 0.05) (Figure 2(d)). Combined with the above results, we confirm that the expression of STRs and associated signaling components was downregulated in renal tissue of DM mouse models.

### 3.3. High Glucose Inhibited the Expression of STRs and Primed NLRP3 Inflammasome Signaling

**Figure 3:**
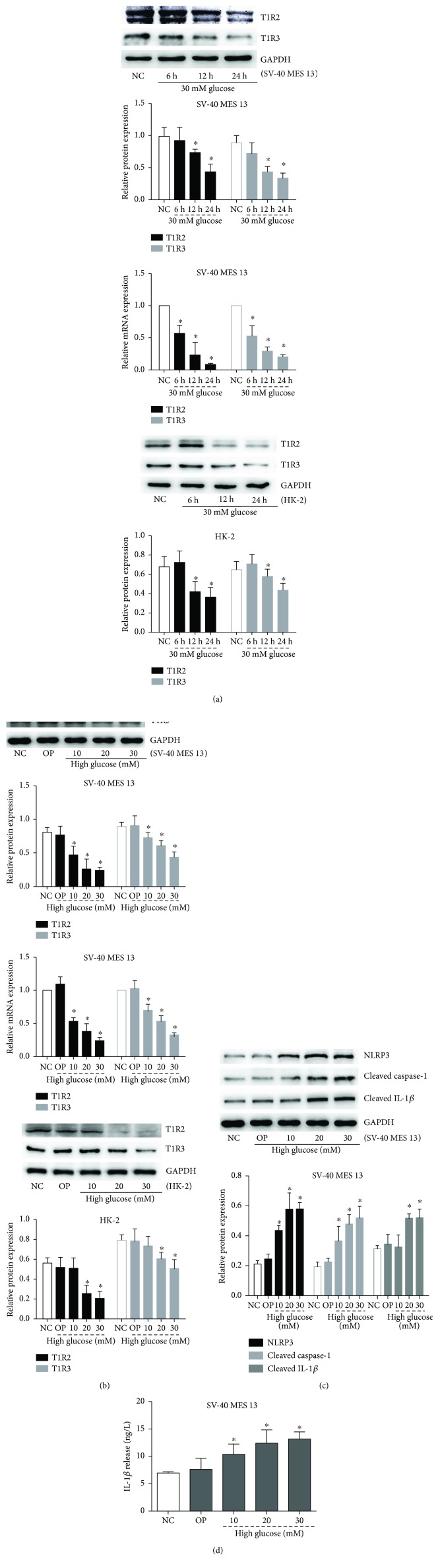
High glucose inhibited the expression of STRs and activated NLRP3 inflammasome. (a) GMCs (SV-40 MES 13) and human proximal tubular cells (HK-2) were treated with 30 mmol/L high glucose for 6, 12, and 24 h, and Western blot or qRT-PCR was performed to detect T1R2 and T1R3 protein or mRNA expression levels. (b) Cells were treated with the indicated concentrations of glucose or mannitol for 24 h. Western blot or qRT-PCR was performed to detect T1R2 and T1R3 protein or mRNA expression levels. (c) Western blotting analysis of NLRP3, cleaved caspase-1, and cleaved IL-1*β* in GMCs treated with an indicated concentration of glucose or mannitol for 24 h. (d) IL-1*β* protein level in the GMC culture supernatant was determined by ELISA. Data are expressed as mean ± SD. ^∗^*p* < 0.05 versus NC group.

To determine whether STRs are regulated by glucose in renal innate cells, we first detected T1R2 and T1R3 expression in GMCs (SV-40 MES 13) and human proximal tubular cells (HK-2). Compared with the NC group, both the protein and mRNA levels of T1R2 and T1R3 in SV-40 MES 13 cells were also significantly inhibited following 6, 12, and 24 h of exposure to 30 mmol/L high glucose in a time-dependent manner (*p* < 0.05); the same trend is found in HK-2 cells (*p* < 0.05) (Figure 3(a)). Similarly, the expression of T1R2 and T1R3 in SV-40 MES 13 cells and HK-2 cells was also significantly decreased by different concentrations of high glucose in a dose-dependent manner (*p* < 0.05) (Figure 3(b)). However, both the protein and mRNA levels of NLRP3, cleaved caspase-1, and cleaved IL-1*β* were also significantly induced by high glucose in a dose-dependent manner (*p* < 0.05) (Figure 3(c)). Meanwhile, high glucose induced inflammatory cytokine IL-1*β* release from SV-40 MES 13 cells in a dose-dependent manner (*p* < 0.05) (Figure 3(d)). These results suggested that high glucose inhibited the expression of STRs and activated NLRP3 inflammasome in *vitro.*

### 3.4. Lactisole Reversed High-Glucose-Induced Downregulation of STRs

**Figure 4:**
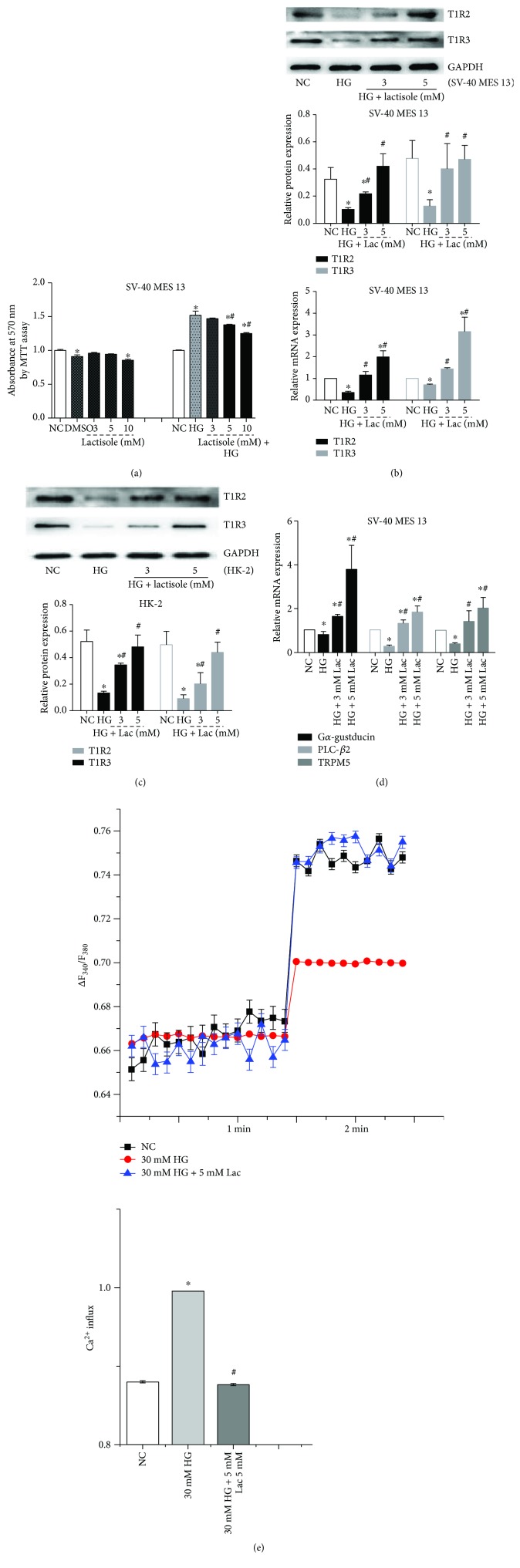
High glucose-induced downregulation of STRs and associated signaling components was reversed by lactisole. Mouse GMCs (SV-40 MES 13) was firstly incubated with 3, 5, and 10 mM lactisole; then 30 mM high glucose (HG) alone was intervened by the indicated concentrations of lactisole following 30 mM high glucose for 24 h. (a) Cell viability was determined by MTT analysis. (b) The protein and mRNA expression of T1R2 and T1R3 in GMCs were detected by Western blotting and qRT-PCR. (c) The protein expression of T1R2 and T1R3 in human proximal tubular cells (HK-2) was detected by Western blot. (d) STR-associated signaling components (*α*-gustducin, PLC*β*2, and TRPM5) were detected by qRT-PCR. (e) GMCs were stimulated with 30 mmol/L glucose in the presence (blue), and absence (red) of 5 mmol/L lactisole, and the Ca^2+^ influx changes in Ca^2+^ dye fluorescence was monitored. Data are expressed as mean ± SD. ^∗^*p* < 0.05 versus NC group. ^#^*p* < 0.05 versus 30 mM glucose (HG) stimulation group.

Lactisole, a STR inhibitor, was used to stimulate GMCs. The effect of different concentrations of lactisole on GMCs' viability was assessed by MTT. We observed that 3–5 mM concentration range of lactisole has no effect on the cell viability (*p* > 0.05) and reversed 30 mM high-glucose-induced proliferation in a dose-dependent manner (*p* < 0.05). However, higher concentrations of lactisole (≥10 mM) may inhibit cell viability (Figure 4(a)). Considering the cytotoxicity detected by MTT and the pharmacological concentrations of lactisole reported in the literature [13], we chose the 3 mM and 5 mM lactisole as the intervention reagent. As expected, high-glucose-inhibited T1R2 and T1R3 expression in SV-40 MES 13 cells (Figure 4(b)) and HK-2 cells (Figure 4(c)) was obviously reversed by lactisole in a dose-dependent manner (*p* < 0.05). qRT-PCR showed that high-glucose-inhibited G*α*-gustducin, PLC*β*2, and TRPM5 were obviously reversed by lactisole in a dose-dependent manner (*p* < 0.05) (Figure 4(d)). Moreover, the intracellular calcium level was measured by using a fluorescent Ca^2+^ indicator Fura-PE3 AM. The results showed that 30 mmol/L glucose stimulation for 24 h induced the decrease of Ca^2+^ after a certain lag period (*p* < 0.05). The intracellular calcium level inhibited by high glucose was significantly reversed by lactisole (*p* < 0.05) (Figure 4(e)). These results suggested that high-glucose-induced downregulation of STRs was reversed by lactisole.

### 3.5. Lactisole Reversed High-Glucose-Induced Activation of ROS-NLRP3 Inflammasome Signaling

**Figure 5:**
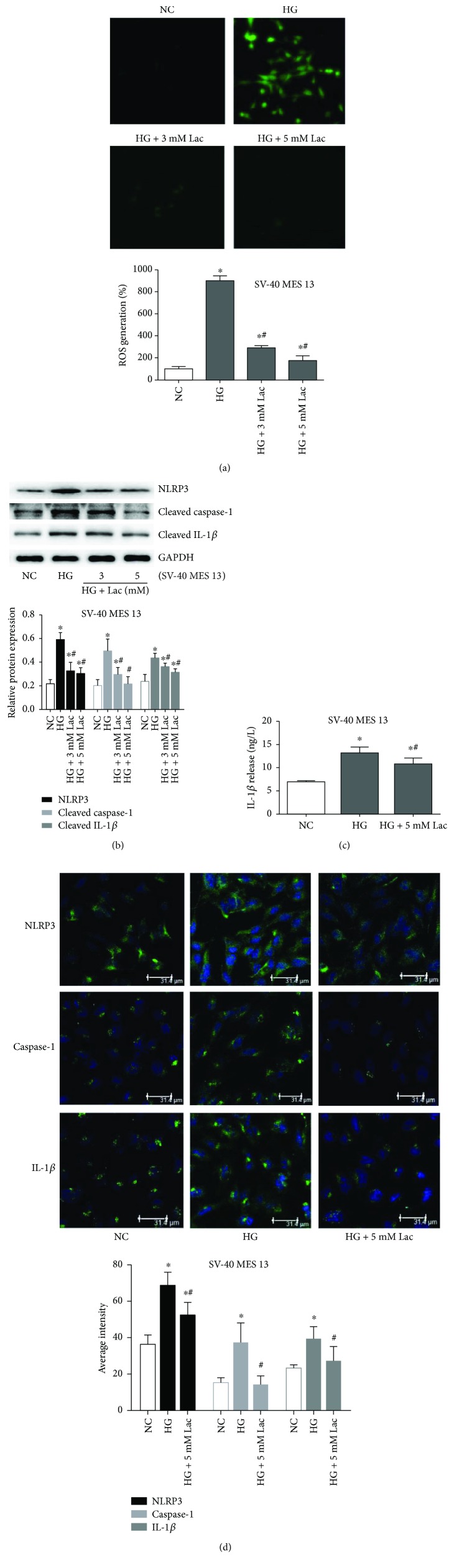
High glucose-induced activation of ROS-NLRP3 inflammasome signaling was reversed by lactisole. (a) The fluorescence images (100×) and quantitative assay for intracellular ROS was determined by detection kits. (b) The protein and mRNA expression of NLRP3, cleaved caspase-1, and cleaved IL-1*β* were detected by Western blotting. (c) IL-1*β* protein level in the cell culture supernatant was assayed after lactisole treatment. (d) Immunofluorescence and a laser scanning confocal microscope (400×) detected the expressions of NLRP3, caspase-1, and IL-1*β* in GMCs as green fluorescence that overlapped with blue fluorescence emitted by the nuclear stain DAPI. The values of semiquantitative analysis for average intensity were assessed and the gray graphs confirmed these trends. Data are expressed as mean ± SD. ^∗^*p* < 0.05 versus NC group. ^#^*p* < 0.05 versus 30 mmol/L glucose (HG) stimulation group.

Next, we assessed whether STRs were involved in the high-glucose-induced ROS-NLRP3 inflammasome signaling in SV-40 MES 13 cells. The results showed that high-glucose-induced intracellular ROS production (Figure 5(a)) and the expression of NLRP3, cleaved caspase-1, and cleaved IL-1*β* (Figure 5(b)) were also abrogated after lactisole treatment in a dose-dependent manner (*p* < 0.05). Meanwhile, high-glucose-induced inflammatory cytokine IL-1*β* release from SV-40 MES 13 cells was blunted by lactisole (*p* < 0.05) (Figure 4(c)). Consistent with this finding, we used immunofluorescence to detect the expression level of NLRP3, caspase-1, and IL-1*β*, and we observed the same changes (*p* < 0.05) (Figure 5(d)). These results suggested that STRs signaling is required for high-glucose-induced ROS-NLRP3 inflammasome signaling activation.

## 4. Discussion

Although inflammatory responses are a critical component in defense against pathogens and damage-associated molecular patterns, too much inflammation is harmful. The molecular underpinnings of this inflammation include nutrient excess-mediated activation of the innate immune NLRP3 inflammasome. How NLRP3 inflammasome formation is triggered remains debatable because several models have been proposed, all of which explain some but not all observations [14, 15]. We reported previously that high glucose and lipopolysaccharide activate ROS-TXNIP-NLRP3 inflammasome signaling in GMCs, but ROS inhibitor N-acetylcysteine (NAC) could not completely inhibit the activation of NLRP3 inflammasome induced by high glucose, suggesting that there may be other pathways by which high glucose primes ROS-NLRP3 inflammasome signaling [16].

A recent paradigm in sensory physiology suggests that several classes of understudied receptors (olfactory receptors (ORs), taste receptors, and orphan GPRs) play key roles in nonsensory tissues, where they serve as selective and sensitive chemoreceptors [17, 18]. Previous research showed that the intestinal STRs, T1R2 and T1R3, were expressed in distinct epithelial cells in the human proximal intestine and that their transcript levels varied with glycemic status in patients with type 2 diabetes [11, 19]. Recent research found that STRs were also expressed in extragustatory organs regulating carbohydrate metabolism. T1R2 and T1R3 are expressed in pancreatic *β*-cells; inhibition of this receptor by gurmarin or deletion of the T1R3 gene attenuates glucose-induced insulin secretion from *β*-cells [9, 13]. However, few reports exist on the regulation of taste receptors in renal tissues under the condition of diabetes. Just as ORs were found in a variety of additional tissues including the kidney and considered as a novel mechanism of blood pressure regulation and kidney glucose handling [20], here, our research indicated that STRs and associated signaling components were also expressed in kidney in vivo and in vitro; furthermore, we characterize for the first time that compared with the NC group, these signaling molecule expressions were downregulated in DM group, suggesting their modulation under the condition of diabetes.

It is becoming increasingly clearer that taste receptors have important roles beyond simply that of gustation. Studies have shown that taste buds express various molecules involved in innate immune responses, including the proinflammatory cytokine tumor necrosis factor (TNF), suggesting that TNF signaling preferentially modulates bitter taste responses [21]. Recently, it is thought that the taste receptors have emerged as key players in the regulation of innate immune defenses in the mammalian respiratory tract [22]. These responses are quick in onset and are complementary to traditional antimicrobial pathways, such as those involving TLRs [23]. Dysfunction or genetic variation of bitter or sweet taste receptors appears to play a key role in respiratory disease and associated with some chronic inflammatory diseases. The chemosensory functions of taste receptors as sentinels of innate immunity may partly explain why these receptors are so widely expressed throughout the body [24]. Although the role of STRs in respiratory tract involved in innate immune defenses has been widely studied and information on the presence and possible role of STRs in kidney is relatively scarce, it remains unclear whether they are known to be modulated by glucose level, let alone whether any taste reception is involved in the regulation of inflammatory cytokines.

Our current aims were, therefore, to investigate whether STRs and associated signaling component expression will be changed after long-term treatment with high glucose in renal innate cells (mouse GMCs and human proximal tubular cells (HK-2)). The present study has also shown that the expression of STRs and associated signaling components (G*α*-gustducin, PLC*β*2, and TRPM5) was rapidly downregulated upon exposure to glucose in a time- and dose-dependent manner in vitro. GMC proliferation and hypertrophy, ECM accumulation, as well as consequent renal fibrosis induced by high glucose, AGEs, or LPS, have been recognized as major pathogenic events in the progression of renal failure in DN [25]. Accordingly, our study found 30 mmol/L glucose stimulation for 24 h induced decrease of Ca^2+^ after a certain lag period; meanwhile, NLRP3 inflammasome signaling molecules and inflammatory cytokine IL-1*β* release from GMCs were induced by high glucose, suggesting that STRs could be involved in the activation of NLRP3 inflammasome under high-glucose stress.

It is quite likely that a major component of the STRs may be a homodimer of T1R3 [26]. To block the sweet-sensing receptor, the putative compound should inhibit the homodimer of T1R3. Among various inhibitors of sweet taste perception, lactisole, a broad-acting sweet antagonist, suppresses the sweet taste of sugars, protein sweeteners, and artificial sweeteners and is the most interesting candidate since it acts on the specific region of T1R3 [27]. Lactisole is commercially available and may be useful to assess the function and physiological role of the STRs [28]. The present study was conducted to examine whether or not lactisole can be used as an inhibitor of the glucose-sensing receptor. Our data revealed that 5 mM lactisole reversed the high glucose-inhibited STR expression and intracellular calcium level but blocked high glucose-induced intracellular ROS production and NLRP3 inflammasome signaling activation in a dose-dependent manner in mouse GMCs, suggesting that STR-induced signaling pathway may be involved in the regulation of innate immune, and 5 mM lactisole may be useful in assessing the role of the glucose-sensing receptor in mouse GMCs. However, it must be pointed out that the mechanism by which STRs regulate the activation of ROS-NLRP3 inflammasome is not clear, and the specific impact that this modulation of sweet taste signaling has on the development of DN still has to be elucidated. Furthermore, our studies by no means rule out other potential mechanisms by which STRs may mediate oxidative stress and inflammation. Recent research has already showed that the T1R1/T1R3, as a direct sensor of the fed state and amino acid availability, which was found in most tissues, could activate mTOR and inhibit autophagy [29, 30]. In view of mTOR and autophagy involved in the regulation of NLRP3 inflammasome, we speculate that STRs may be involved in the regulation of ROS-NLRP3 inflammasome signaling by modulating mTOR and autophagy. Even so, our study shows that a direct role of the kidney in sweet taste sensing and subsequent regulation of ROS-NLRP3 inflammasome signaling should not be excluded and deserves further research.

## 5. Conclusion

Our study confirms that high glucose inhibited the expression of STRs and associated signaling components and primed ROS-NLRP3 inflammasome signaling in vivo and in vitro. STR inhibitor lactisole reversed high-glucose-induced activation of ROS-NLRP3 inflammasome signaling. Our study supports the hypothesis that high glucose induced ROS-NLRP3 inflammasome signaling activation in part via STRs, suggesting that STRs may act as new therapeutic targets of DN.

## Figures and Tables

**Table 1:** Primer sequences for qRT-PCR.

Gene	Forward sequence	Reverse sequence
T1R2	5′-GTCCGCTGCACCAAGCA-3′	5′-GTTCGTCGAAGAAGAGCTGGTT-3′
T1R3	5′-AGCTTCTTCCTCATGCCACA-3′	5′-GCCATAGTCATCATCACTCCCT-3′
G*α*-gustducin	5′-TGCATTATATTTTGCGCAGC-3′	5′-AGAACAATGGAGGTGGTTGC-3′
PLC*β*2	5′-TGGTAGCCACCACCCTTTCCATTA-3′	5′-AGTTCCACTTCCACATAGGTGCGT-3′
TRPM5	5′-CCAGCATAAGCGACAACATCT-3′	5′-GAGCATACAGTAGTTGGCCTG-3′
NLRP3	5′-AGAAGAGACCACGGCAGAAAG-3′	5′-CTTGGAACCAGGTTGAGTGT-3′
Caspase-1	5′-TGGAAGGTAGGCAAGACT-3′	5′-ATAGTGGGCATCTGGGTC-3′
IL-1*β*	5′-GTCTTTCCCGTGACCTTC-3′	5′-ATCTCGGAGCCTGTTAGTGC-3′
*β*-Actin	5′-ACCTCTATGCCAACACAGTG-3′	5′-GGACTCATCGTACTCCTGCT-3′
